# Diagnosis and subtype analysis of *Blastocystis* sp*.* in 442 patients in a hospital setting in the Netherlands

**DOI:** 10.1186/1471-2334-13-389

**Published:** 2013-08-23

**Authors:** Aldert Bart, Ellen MS Wentink-Bonnema, Henk Gilis, Nienke Verhaar, Carla JA Wassenaar, Michèle van Vugt, Abraham Goorhuis, Tom van Gool

**Affiliations:** 1 Parasitology Section, Department of Medical Microbiology, Center for Infection and Immunity Amsterdam (CINIMA), Academic Medical Center, Amsterdam, The Netherlands; 2 Center for Tropical and Travel Medicine, Department of Infectious Diseases, Academic Medical Center, University of Amsterdam, Amsterdam, The Netherlands; 3 Infectious Diseases Epidemiological Unit, Public Health School, Université Libre de Bruxelles, Brussels, Belgium

**Keywords:** *Blastocystis*, Diagnosis, Microscopy, PCR, Molecular epidemiology

## Abstract

**Background:**

*Blastocystis* sp*.* are among the most commonly observed intestinal parasites in routine clinical parasitology. *Blastocystis* in humans consists of at least 9 genetic subtypes. Different subtypes of *Blastocystis* may be associated with differences in pathogenicity and symptomatology.

**Methods:**

Advanced microscopy on two samples and sequence-confirmed PCR on a third sample from the same individual were used for *Blastocystis* diagnosis and subtype analyses on routine clinical samples in a university hospital.

**Results:**

With a combined gold standard of sequence-confirmed PCR and positive advanced microscopy, 107 out of 442 (24.2%) patients were diagnosed with *Blastocystis*. infection, which is a high frequency of detection in comparison to previous reports from industrialized countries. The sensitivity of microscopy and sequence-confirmed PCR was 99.1% (106/107) and 96.3% (103/107), respectively.

Among 103 typable samples, subtype 3 was most abundant (n = 43, 42%), followed by subtypes 1 and 2 (both n = 23, 22%), subtype 4 (n = 12, 12%), and single samples with subtypes 6 (1%) and subtype 7 (1%). The prevalence of *Blastocystis* infection was 38% in patients from the Department of Tropical Medicine and 18% in patients from other departments.

**Conclusions:**

A high prevalence of *Blastocystis* infection was found with both advanced microscopy and sequence-confirmed PCR in our patient population. Most cases were caused by subtypes ST1, ST2, ST3 and ST4. A significantly higher prevalence was found among patients with a history of recent travel to tropical countries.

## Background

*Blastocystis* is one of the most prevalent unicellular parasites in human faecal specimens. The prevalence of *Blastocystis* has been reported to range from 60% in tropical, subtropical and developing countries [1] to as low as 0.5% in Japan [2]. Considerable variation in reported prevalence can be related to the epidemiological setting, especially the patient population studied, but is probably also due to differences in the diagnostic methods used [3]. Transmission of *Blastocystis* is suggested to be by the faecal-oral route, e.g. via contaminated water or food, with sources being both humans and animals [4-6].

Clinical features of *Blastocystis* infection are reported to include nausea, anorexia, abdominal pain, flatulence and acute or chronic diarrhoea [7,8]. In addition, an association is suggested with symptomatology in irritable bowel syndrome [9-13] and, as an opportunistic pathogen, in immunocompromised patients [14,15]. Other studies however suggest *Blastocystis* to be a commensal parasite in humans without any pathogenic role [16-18].

Conflicting views about pathogenicity may be related to the number of infecting parasites present, duration of infection (acute or chronic), host genetic factors or to different subtypes or species of *Blastocystis* infecting humans [3,19-27]. Currently 17 subtypes or species of *Blastocystis* have been described based on sequence analysis, of which 9 have been reported in humans [28-31].

For proper study of prevalence, epidemiology and pathogenic potential of *Blastocystis*, reliable and practical diagnosis is of paramount importance. Techniques currently in use include microscopy, molecular detection, and xenic *in vitro* culture [3]. Recent studies suggest certain molecular methods to have highest sensitivities [26,32-34]. In this report we compare sequence-confirmed PCR to advanced microscopy, and describe the prevalence of *Blastocystis* infection and subtype determination in a large series of patients in The Netherlands.

## Methods

### Samples and parasitological examination

Stool samples of 442 patients submitted for routine parasitological examination at the Department of Clinical Parasitology, Academic Medical Center (AMC) were collected and processed with the Triple Faeces Test (TFT) as described previously [35]. Microscopy with the TFT has been part of routine clinical care in the AMC for 18 years. In brief, with the TFT every patient fills three tubes with stools on 3 consecutive days. Tubes of day 1 and day 3 are prefilled with SAF (sodium acetate–acetic acid–formalin), while the tube of day two is empty. After 3 days the complete TFT set is sent to the laboratory by the patient. Compliance of patients for proper delivery of the whole set is high [35]. SAF-stool suspensions of days 1 and 3 are studied in the laboratory by an iodine stained wet smear and, in case of undetermined protozoa, by a permanent Chlorazol Black stain [35]. Fresh (non-fixed) stool of day 2 is routinely processed by the formol ether concentration technique (FECT) for diagnosis of (cysts and eggs of) protozoa and helminths. In addition, on sample 2 auramine stain for *Cryptosporidium*, Uvitex 2B for Microsporidia*,* antigen tests and PCR are performed when indicated. In this study PCR for *Blastocystis* was performed on sample 2.

For microscopic diagnosis of *Blastocystis* from each SAF sample approximately 10 μl of SAF stool sample was mixed with approximately 20 μl of Lugol’s iodine solution and covered with a 21 × 26 mm cover slip. The slide was examined for intestinal parasites, including *Blastocystis* for 300 optical fields with a magnification of 250× (20× objective and 12.5 eye piece) and, in case of suspected organisms, 500 × (40 × 12.5×). Duration of this examination per slide is 5 minutes on average. The diagnostic criterion for positivity of *Blastocystis* species was at least 2 clear vacuolar forms of the parasite in either of the two SAF samples. Granular or amoeboid stages of *Blastocystis* species were not studied.

Permanent staining with Chlorazol Black was routinely performed on any SAF sample where further determination was needed, as is true for *Dientamoeba fragilis* and other vegetative stages of protozoa. Results of iodine wet smear and Chlorazol Black stain were compared on a selected series of positive and negative samples. Technicians performing microscopic diagnosis had extensive experience in parasitological diagnosis and were blinded for the results of PCR.

### Molecular methods

All non-fixed stools collected on the second day were mixed with Stool Transport and Recovery (S.T.A.R.) Buffer (Roche Applied Science) to an approximately 1:3 v:v suspension and vortexed and stored for DNA isolation and PCR. DNA was isolated from all faecal suspensions using the MagNA Pure LC Isolation instrument for nucleic acid extraction (Roche Applied Science). To monitor the process of extraction and amplification, a universal control consisting of phocine herpesvirus type 1 (PhHV-1) was added to the clinical specimens [36]. Briefly, 20 μl of faecal suspension was added to 500 μl lysis buffer with 2500 copies PhHV-1 and prelysed for 10 minutes at room temperature. After centrifugation, 500 μl of the lysate supernatant was used as input for the Total Nucleic Acid Isolation Kit (Roche Applied Science) as described by the manufacturer. Elution of nucleic acid was in a final volume of 50 μl. *Blastocystis* PCR was performed using primers and conditions described by Stensvold [33], aimed at a region of the small subunit rRNA gene of *Blastocystis*, using 15 μl of isolated DNA as input in a total volume of 50 μl. Fifteen microliters of PCR product were size-separated by agarose gel electrophoresis and visualized by UV illumination after ethidium bromide staining. Diluted amplicons were sequenced on both strands using amplification primers with BigDyeTerminator chemistry (Applied Biosystems) and analysed on an ABI 3900 sequencer. Subtypes following the nomenclature proposed by Stensvold et al [21] were determined by comparison of obtained sequences to Genbank reference sequences using CodonCode Aligner program (CodonCode Corporation), and MEGA 4.0 [37]. For sequences that seemed to contain a mixed signal of multiple subtypes, the predominant subtype was assigned. Only samples for which a *Blastocystis* sequence was obtained were considered PCR and sequencing positive. Samples for which no *Blastocystis* sequence was obtained or for which there was no PCR amplicon while the PhHV-1 control DNA was detected using real-time PCR [36], were considered negative. PCR and sequence interpretation were performed blinded to the microscopy results. Sequences were deposited in Genbank with accession numbers KF241991-KF242090.

### Database and statistics

Samples and patient data (sex, age, referring department) were anonymized and aggregated in a database. For evaluation of diagnostic tests using anonymized data no medical-ethical approval is required in The Netherlands, provided that patients declare no objection to such use of their samples. Computations of *P* values using Pearson’s χ^2^ test were performed at VassarStats website (http://vassarstats.net/index.html). *P* values below or equal to 0.05 were regarded as significant.

## Results

### Study population and microscopy

From October 2008 to January 2009, 442 TFT sets from 442 patients were included in the study. The 442 patients comprised 230 females (median age 29, range 1-82) and 212 males (median age 34, range 0-87 years). Patients were referred by the Tropical Diseases Department (133), Emma Children’s Hospital (103), general practicioners (70), Department of Internal Medicine (63), Department of Gastroenterology and Hepatology (45), or other departments (28).

In 148 out of 442 (33%) patients one or more intestinal parasitic species were observed. Protozoa were observed in 148 out of 442 (33%) and helminths in 3 out of 442 (0.7%) patients.

*Blastocystis* were recognized in either of the two SAF preserved samples by microscopy of iodine stained wet smears in 106 out of 442 (24.0%) patients in any of two samples. In 90 patients, both SAF samples (day 1 and day 3) were positive for *Blastocystis*. In 10 patients *Blastocystis* was observed only in the sample of the first day, and in 6 only in the sample of the third day. From 106 patients positive for *Blastocystis* with iodine wet smear, 34 times a permanent Chlorazol Black stain was performed for determination of other protozoa. In all cases *Blastocystis* were also observed with the Chlorazol Black stain. From 336 patients negative for *Blastocystis* with iodine wet smear, 81 times a permanent stained slide also was studied. In none of these *Blastocystis* was observed.

### PCR and *Blastocystis* subtype determination

In 103 out of 442 (23.3%) patients, sequence-confirmed PCR was positive. Six subtypes were observed among the 103 typable samples: subtype 3 was most abundant (n = 43, 41%), followed by subtypes 1 and 2 (both n = 23, 22%), subtype 4 (n = 12, 12%), and single samples with subtypes 6 (1%) and subtype 7 (1%) (Table 1).

**Table 1:** ***Blastocystis *infection status of patients admitted via the Department of Tropical Medicine and patients admitted via other departments**

**Patient samples**	**Total patients examined**	***Blastocystis*positive (%)**	***ST1***	***ST2***	***ST3***	***ST4***	***Other ST***	***NST***^***a***^
Submitted via dept tropical diseases	133	50 (38%)^b^	13 (26%)	10 (20%)^c^	20 (40%)	5 (10%)	1 (2%)	1 (2%)
Submitted via other departments	309	57 (18%)^b^	10 (18%)	13 (23%)	23 (40%)	7 (12%)	1 (2%)	3 (5%)
Total population	442	107 (31%)	23 (21%)	23 (21%)	43 (40%)	12 (11%)	2 (2%)	4 (4%)

^a^ NST: not subtypable. These isolates were positive by microscopy only.

^b^ p < 0.0001.

^c^ one of these patients was negative by microscopy.

### Gold standard, congruence between microscopy and PCR

Assuming 100% specificity of both microscopy and sequence-confirmed PCR, positive findings of both tests were combined to serve as a gold standard. Using this standard, 107 of 442 patients (24.2%) were positive for *Blastocystis*. Microscopy, with reading of two SAF samples studied per patient, had a sensitivity of 99.1% (106/107). Sequence-confirmed PCR, performed on stool of the day 2 sample only, had a sensitivity of 96.3% (103/107)*.*

In five samples, microscopy and sequence-confirmed PCR were not in agreement. This concerned 4 times a microscopy positive and sequence-PCR negative result, and one sequence- PCR positive and microscopy negative result. All five samples were retested with both methods. The four microscopically positive samples that were PCR and sequence negative, were all positive for the PhHV-1 extraction and amplification control, suggesting that PCR was not negative due to insufficient DNA extraction or inhibitory factors in the eluate, but due to the amount of *Blastocystis* DNA being below detection level. This is in accordance with the observation of only few parasites (<5 per total slide) in two of these samples by microscopy. In the other two samples, more *Blastocystis* cells were observed. By agarose gel electrophoresis of PCR products, faint bands were visible in these samples. Apparently, the yield of PCR amplicon was insufficient to obtain good sequences in these two samples. Alternatively, the microscopical observations may be explained by structures that are morphologically highly similar to *Blastocystis*. In the one sample that was PCR and sequencing positive, but negative in microscopy, a weak band was observed in PCR that yielded a sequence. Possibly, only *Blastocystis* DNA or cysts were present in this sample, rather than intact *Blastocystis* cells.

### Relation between *Blastocystis* infection, subtype and patient characteristics

In the patient population from the Department of Tropical Medicine, significantly (p < 0.0001; Pearson’s χ^2^ test) more *Blastocystis* infection was found (38%, 50/133) as compared to patients from other departments (18%, 57/309), as shown in Table 1. There was no significant difference in the subtype distribution between patients presenting at the Department of Tropical Medicine and patients presenting at other departments.

No significant correlation between patient gender and *Blastocystis* infection was found. Patients with *Blastocystis* infection were more frequently coinfected with *Dientamoeba fragilis* (17%, 18/107) than patients without *Blastocystis* infection (8%, 26/335) (p < 0.006; Pearson’s χ^2^ test). No significant correlation of the subtype of *Blastocystis* and *Dientamoeba* infection was observed.

## Discussion

In our hospital-derived study population, 24% (107/442) of patients were infected with *Blastocystis*. This is comparable to the reported 23% prevalence found in a smaller (n = 93 patients) hospital population in Denmark with direct PCR on stool samples [33], and higher than 19% reported in Australia where a combination of microscopy, culture and PCR was used for diagnosis [32]. In similar teaching hospital settings in Italy [38] and the UK [[39]] considerably lower (7%) prevalence was observed using microscopical methods on one unfixed stool specimen for diagnosis. The higher prevalence in our study can be due to selection of our hospital population, assuming pathogenicity of *Blastocystis*, but also to improved sensitivity of the diagnostic procedures used.

The high prevalence in our population was observed both by sequence-confirmed PCR and by microscopy. With excellent specificity of both methods, the prevalence observed most likely represents the true occurrence of the parasite in our population. The high sensitivity of microscopy was remarkable compared to previous studies [32,39,40]. This can be explained by the use of two fixed samples in the Triple Faeces Test rather than single or fresh (non-preserved) samples as used in previous studies [38,39]. In our study, microscopy on two stool samples, compared to one sample, increased the sensitivity of microscopy up to 10%. This is in agreement with earlier reports where intermittent shedding of *Blastocystis* was observed and stresses the need for multiple stool examinations with microscopy before reporting a negative result [41,42]. Moreover, with the Triple Faeces Test patients themselves add stool samples immediately after defaecation to SAF fixative, reducing the time to fixation to a minimum. This is in contrast to earlier reports where stools were added to a fixative only after delivery in the laboratory [32]. In fresh samples, vegetative stages of protozoa, including *Blastocystis*, may rapidly deteriorate and become microscopically unrecognizable [43]. Timely fixation therefore is important for high diagnostic sensitivity of *Blastocystis* with microscopy.

Although microscopy on two samples proved very effective for diagnosis of *Blastocystis*, equal results with PCR diagnosis were obtained with examination of one sample only. In addition, determination of the subtype can be obtained, which is not possible by microscopy. We included sequence confirmation as a criterion for a positive PCR, to assure 100% specificity. Sequence-confirmed PCR was positive in 103 out of 107 (97%) positive patients. Discrepancies between sequence-confirmed PCR and microscopy in our study probably were related to very low numbers of *Blastocystis* shedded in the stools, resulting in insufficient amounts of PCR product to obtain a sequence.

The subtype distribution in the study population showed six different subtypes: ST1, ST2, ST3, ST4, ST6 and ST7. Subtypes 1-4 have been found as the most common subtypes in previous studies as well, [19,22,44-49], including molecular studies using samples initially cultured in vitro. Various studies have identified ST3 as the predominant subtype, as was true for our study population. Subtype 4 was relatively frequent in our study group (12%) as compared to studies worldwide [50], although a more similar prevalence of this subtype was observed in studies from Europe [25]. The differences in relative abundance of the subtypes 1-4 suggests that differences in transmission efficiency may exist between them. These could include differences in cyst numbers, cyst survival, exposure to cysts, infectious dose or transmission cycles. The single subtype 6 and subtype 7 cases may reflect zoonotic infections, as these subtypes have been reported in stools from birds [51,52].

The significantly higher prevalence of *Blastocystis* infection in patients presenting at the Department of Tropical Medicine as compared to other departments, suggests that travel to tropical countries can increase the risk of *Blastocystis* infection, as has been observed in earlier studies [7,8,53,54]. High prevalence of *Blastocystis* has been reported in developing countries [1,44,55,56]. Despite the higher prevalence, the *Blastocystis* subtype distribution among travelers and patients of other clinical departments was similar. This suggests that general poor hygiene and sanitation in many of the tropical countries rather than specific exposure to certain *Blastocystis* subtypes, causes the observed increase in prevalence. Unfortunately, no specific data about previous travel to tropical countries were available for the patients from departments other than the Department of Tropical Medicine.

## Conclusions

In conclusion, fast and reliable diagnosis of *Blastocystis* infection in routine clinical parasitology is possible by advanced microscopy on multiple fixed stool samples with immediate fixation after production of stools. In terms of sensitivity, specificity and subtype determination, direct PCR on a single stool sample is also an excellent diagnostic tool. The prevalence of *Blastocystis* infection and the ongoing debate about pathogenicity warrant the use of good diagnostic methods in routine clinical laboratories. Other subjects of interest are patients with gastrointestinal complaints returning from the tropics without pathogenic microorganisms but only *Blastocystis* in stools. Further studies on pathogenicity, and especially the importance of subtypes of *Blastocystis*, are needed, as well as identification of an effective treatment. For many of these studies excellent laboratory diagnosis is a prerequisite.

## Competing interests

The authors declare that they have no competing interests.

## Authors’ contributions

AB designed the study, analyzed data and drafted the manuscript, EMSW-B and CJAW collected and analyzed advanced microscopy data, HG and NV collected and analyzed PCR and sequence data, MvV and AG collected patient data and helped in drafting the manuscript, TvG conceived, designed and coordinated the study and drafted the manuscript. All authors read and approved the final manuscript.

## Pre-publication history

The pre-publication history for this paper can be accessed here:

http://www.biomedcentral.com/1471-2334/13/389/prepub
